# The Gβ1 and Gβ3 Subunits Differentially Regulate Rat Vascular Kv7 Channels

**DOI:** 10.3389/fphys.2019.01573

**Published:** 2020-01-14

**Authors:** Iain A. Greenwood, Jennifer B. Stott

**Affiliations:** Vascular Biology Research Centre, Institute of Molecular and Clinical Sciences, St George’s University of London, London, United Kingdom

**Keywords:** G protein βγ subunits, Kv7 channels, vascular smooth muscle, G protein signaling, potassium channel

## Abstract

Within the vasculature Kv7 channels are key regulators of basal tone and contribute to a variety of receptor mediated vasorelaxants. The Kv7.4 isoform, abundant within the vasculature, is key to these processes and was recently shown to have an obligatory requirement of G-protein βγ subunits for its voltage dependent activity. There is an increasing appreciation that with 5 Gβ subunits and 12 Gγ subunits described in mammalian cells that different Gβ_*x*_γ_*x*_ combinations can confer selectivity in Gβγ effector stimulation. Therefore, we aimed to characterize the Gβ subunit(s) which basally regulate Kv7.4 channels and native vascular Kv7 channels. In Chinese Hamster Ovary cells overexpressing Kv7.4 and different Gβx subunits only Gβ1, Gβ3, and Gβ5 enhanced Kv7.4 currents, increasing the activation kinetics and negatively shifting the voltage dependence of activation. In isolated rat renal artery myocytes, proximity ligation assay detected an interaction of Kv7.4 with Gβ1 and Gβ3 subunits, but not other isoforms. Morpholino directed knockdown of Gβ1 in rat renal arteries did not alter Kv7 dependent currents but reduced Kv7.4 protein expression. Knockdown of Gβ3 in rat renal arteries resulted in decreased basal K^+^ currents which were not sensitive to pharmacological inhibition of Kv7 channels. These studies implicate the Gβ1 subunit in the synthesis or stability of Kv7.4 proteins, whilst revealing that the Gβ3 isoform is responsible for the basal activity of Kv7 channels in native rat renal myocytes. These findings demonstrate that different Gβ subunits have important individual roles in ion channel regulation.

## Introduction

The Kv7 family of voltage gated potassium channels are crucial regulators of membrane excitability (Haick and Byron, 2016; Barrese et al., 2018). Within the vasculature this role extends itself to regulating cell contractility where the activity of these channels subdues the activity of voltage gated calcium channels and thus the entry of Ca^2+^− the primary contractile stimulus. This has been evidenced in several vessel types where pharmacological blockade of Kv7 channels results in increased contractility of vessels in *ex vivo* preparations (Chadha et al., 2012; Lee et al., 2015; Stott et al., 2015), and reduced renal blood flow *in vivo* (Salomonsson et al., 2015). Of the 5 Kv7 isoforms (Kv7.1–Kv7.5), the Kv7.4 channel has been most implicated in the regulation of vascular reactivity – molecular knockdown of this isoform increases vessel contractility whilst decreasing the ability to respond to vasodilators (Chadha et al., 2012, 2014; Stott et al., 2016, 2018), and it is Kv7.4 expression which is reduced in hypertensive animal models (Jepps et al., 2011). Due to their importance in vascular reactivity, uncovering mechanisms which govern native vascular Kv7 channel activity has been of key interest and recently G protein βγ subunits (Gβγ) were shown to be crucial regulators of basal Kv7 channel activity (Stott et al., 2015). These two strongly bound proteins were originally identified as components of heterotrimeric G proteins, which couple to G protein-coupled receptors (GPCR). However, Gβγ subunits were acknowledged to be important signaling mediators with the discovery that muscarinic acetylcholine induced hyperpolarization of cardiomyocytes occurred via Gβγ-mediated activation of the K_*ACh*_ channel (Kir3.1/3.4) (Logothetis et al., 1987). Subsequently it has been shown that Gβγ regulate other ion channels [CaV_2_ (Herlitze et al., 1996), TRPM3 (Badheka et al., 2017; Dembla et al., 2017; Quallo et al., 2017)] as well as numerous enzymes (e.g., adenylyl cyclase, PI-3 Kinase (Federman et al., 1992; Stephens et al., 1994; Ford et al., 1998).

Proximity ligation assays (PLA) studies with Kv7.4 and Gβ antibodies revealed a high level of channel-Gβγ interaction in unstimulated smooth muscle cells and structurally different inhibitors of Gβγ effector sites (e.g., gallein, M119K, GRK2i) attenuated heterologously expressed Kv7.4 channels and smooth muscle Kv7 currents in the absence of receptor stimulation (Stott et al., 2015). These findings revealed that Kv7.4 is constitutively regulated by an obligatory interaction with Gβγ. However, there are 5 Gβ (1–5) and 12 Gγ (1–5, 7–13) subunits described in mammals, and subunits display specificity in forming dimer pairs with the Gβ_*x*_γ_*x*_ composition known to alter GPCR behavior and effector coupling (Macrez-Lepretre et al., 1997; Bayewitch et al., 1998a, b; McIntire et al., 2001; Khan et al., 2015). Moreover, there are preferential associations for GPCR-ion channel couplings e.g., Gβγ inhibition of N-type calcium channels after α2-adrenoceptor stimulation is more effective when the receptor is coupled with Gβ1 or Gβ2 (Mahmoud et al., 2012), whilst β1-adrenoceptor coupling to Kir3.2 displays a preference for Gβ5 containing dimers (Robillard et al., 2000). We aimed to determine if a specific Gβ subunit was responsible for the basal regulation of Kv7.4 in native arterial smooth muscle cells and ascertain the effect of the five different Gβ isoforms on heterologously expressed Kv7.4. Our data reveal a striking difference between different Gβ isoforms that impacts on vascular responsiveness.

## Results

**FIGURE 1:**
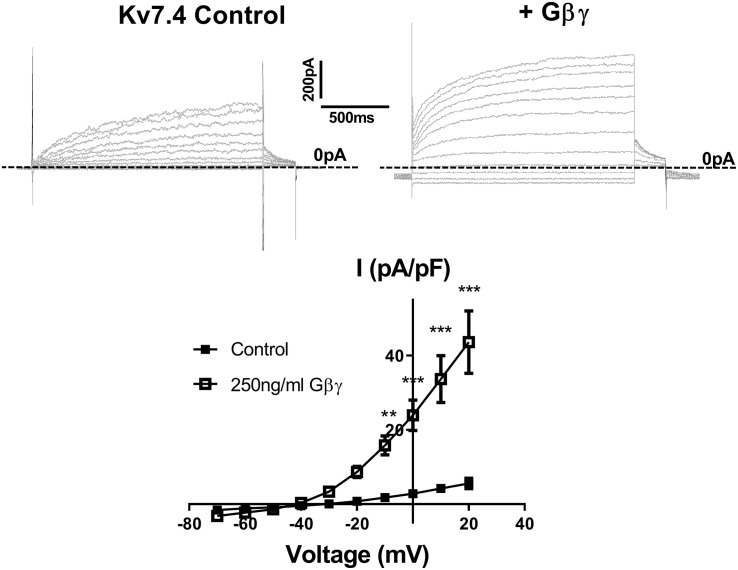
Gβγ enhances Kv7.4 currents in CHO expression system. Top panels show representative traces of currents generated in cells expressing Kv7.4 either with the control intracellular solution (left) or with 250 ng/ml Gβγ purified from bovine brain (right). Zero current is indicated by the dashed line. Mean IV relationship of Kv7.4 currents in control (*n* = 7) or with 250 ng/ml Gβγ (*n* = 4) is shown in bottom panel. Data was analyzed by Bonferroni *post hoc* test following a two-way ANOVA. *p* < 0.01 is denoted (^∗∗^) and *p* < 0.005 is denoted (^∗∗∗^).

**FIGURE 2:**
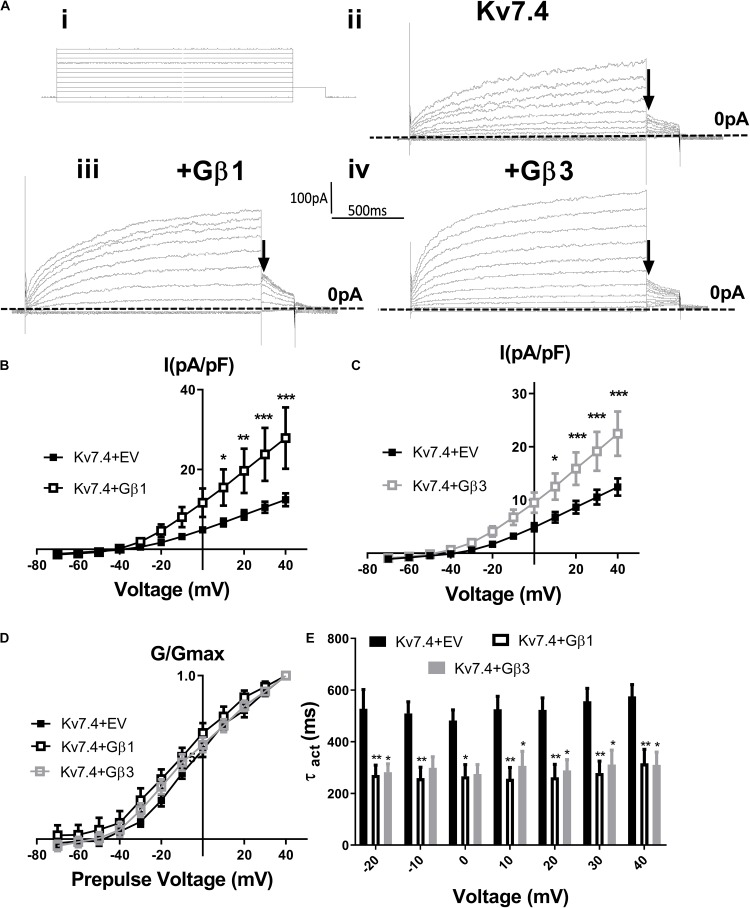
Gβ1 and Gβ3 positively modulate Kv7.4 currents. **(Ai)** Diagram of voltage step protocol used to examine IV relationships. **(Aii–iv)** Representative traces of currents generated in cells expressing Kv7.4 with an empty vector (EV) (ii), Gβ1 (iii) or Gβ3 (iv). Zero current is indicated by the dashed line and point used for determination of voltage dependence of activation is indicated by the arrow. Mean data is shown in panel **(B)** (Gβ1, *n* = 14) and panel **(C)** (Gβ3, *n* = 13). **(D)** Voltage dependence of activation to determine V1/2 of Kv7.4 with EV (−2.4 ± 2.3 mV), Gβ1 (−9.12 ± 3.9 mV), and Gβ3 (−5.9 ± 3.3 mV). **(E)** Time dependence of activation of Kv7.4 currents when expressed with EV, Gβ1, and Gβ3 from −20 mV to 40 mV. Data was analyzed by Bonferroni *post hoc* test following a two-way ANOVA. *p* < 0.05 is denoted (^∗^), *p* < 0.01 is denoted (^∗∗^) and *p* < 0.005 is denoted (^∗∗∗^).

**FIGURE 3:**
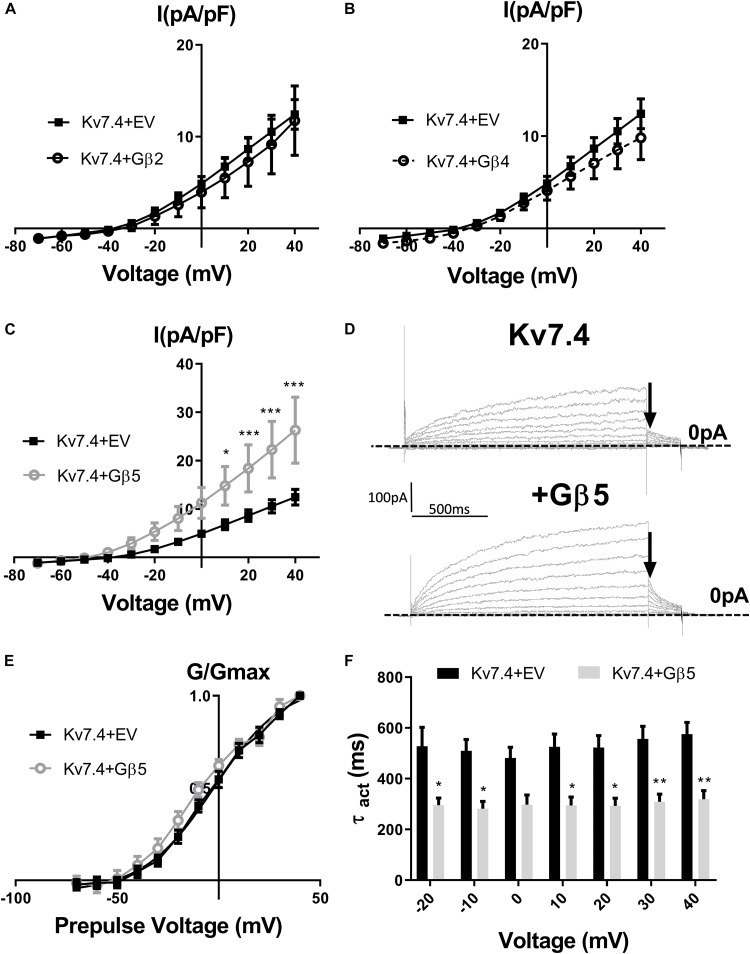
Gβ2, Gβ4, and Gβ5 effects on Kv7.4 currents. IV relationships of Kv7.4 currents in empty vector (EV) or when co-transfected with Gβ2 (**A**, *n* = 12), Gβ4 (**B**, *n* = 10) or Gβ5 (**C**, *n* = 12). **(D)** Representative traces of currents cells expression Kv7.4 + EV (upper panel) or with Gβ5 (lower panel). Zero current is indicated by the dashed line and point used for determination of voltage dependence of activation is indicated by the arrow. **(E)** Voltage dependence of activation curves to determine V1/2 of Kv7.4 with EV (−2.4 ± 2.3 mV) and Gβ5 (−8.1 ± 3 mV). **(F)** Time dependence of activation of Kv7.4 currents when expressed with EV or Gβ5 from −20 mV to 40 mV. Data was analyzed by Bonferroni *post hoc* test following a two-way ANOVA. *p* < 0.05 is denoted (^∗^), *p* < 0.01 is denoted (^∗∗^), and *p* < 0.005 is denoted (^∗∗∗^).

Stott et al. (2015) showed that intracellular perfusion of heterogeneous Gβγ subunits isolated from bovine brain enhances heterologously expressed Kv7.4 currents, produces a leftward shift in the voltage dependence of activation and reduces the rate of activation of these currents. These findings were replicated in CHO cells transiently transfected with Kv7.4, where intracellular perfusion with 250 ng/ml of Gβγ subunits significantly increased voltage dependent currents compared with control (Figure 1). Gβ1–4 show a high degree of homology, whereas Gβ5 is the most structurally distinct Gβ subunit. To determine the effect of individual Gβ subunits from the structurally similar Gβ1–4 group on Kv7.4 currents, we examined the effect each of these subunits on heterologously expressed Kv7.4 channels. Chinese Hamster Ovary (CHO) cells transfected with both Kv7.4 and Gβ1 or Gβ3 plasmids significantly increased K^+^ currents compared to cells expressing Kv7.4 and the empty vector [e.g., 12.4 ± 1.6 pA/pF to 27.9 ± 7.7 (Gβ1) and 22.5 ± 4.2 (Gβ3) at 40 mV] (Figure 2). For both Gβ subunits this effect on Kv7.4 was accompanied by a leftward shift in the voltage dependence of activation [from −2.3 mV to −9.1 mV (Gβ1) and −5.9 mV (Gβ3)] (Figure 2D) and an increase in the rate of activation of the currents over a range of voltages [from 576 ± 45.4 ms to 316.7 ± 53.7 ms (Gβ1) and 310 ± 49.2 ms (Gβ3) at 40 mV] (Figure 2E). These effects on kinetics are analogous to the effect of Gβγ subunits isolated from bovine brain on Kv7.4 (Stott et al., 2015). In contrast, neither Gβ2 nor Gβ4 affected heterologously expressed Kv7.4 currents [11.9 ± 3.8 pA/pF (Gβ2) and 14.4 ± 5.7 pA/pF (Gβ4) at 40 mV] (Figures 3A,B). Interestingly, however, the structurally distinct Gβ5 also significantly enhanced Kv7.4 currents (26.3 ± 6.8 pA/pF at 40 mV), produced a leftward shift in the voltage dependence of activation (−8.1 mV) and increased the rate of activation of the currents (319 ± 34 ms) (Figures 3C–F).

**FIGURE 4:**
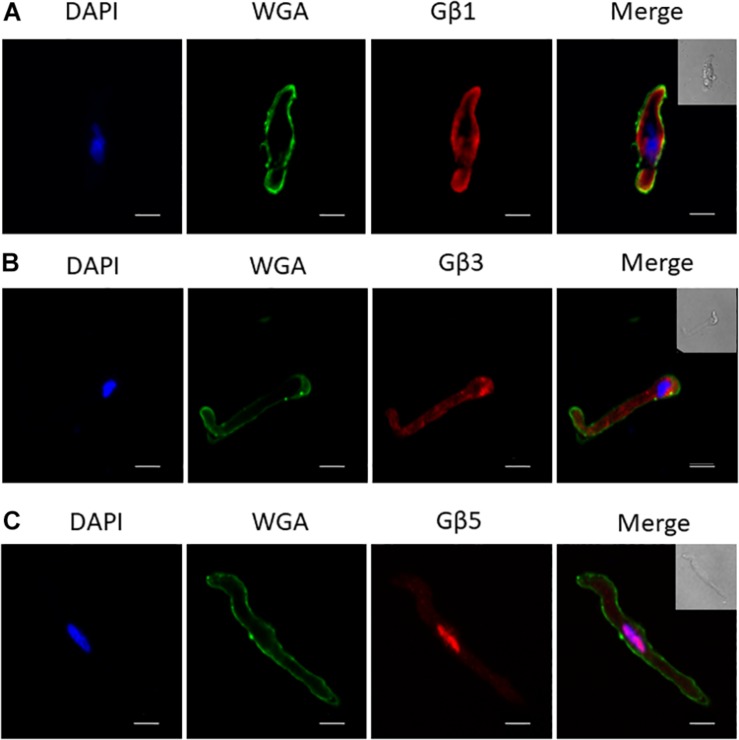
Immunofluorescence of specific Gβ subunits in rat renal artery myocytes. Representative images showing staining in isolated rat renal artery myocytes of Gβ1 **(A)**, Gβ3 **(B)**, and Gβ5 **(C)**, all in red pseudocolour. Nuclear staining by DAPI is shown in blue pseudocolour, while membrane staining by Wheat Germ Agglutinin (WGA) is shown in green pseudocolour. Scale bar: 10 μm.

**FIGURE 5:**
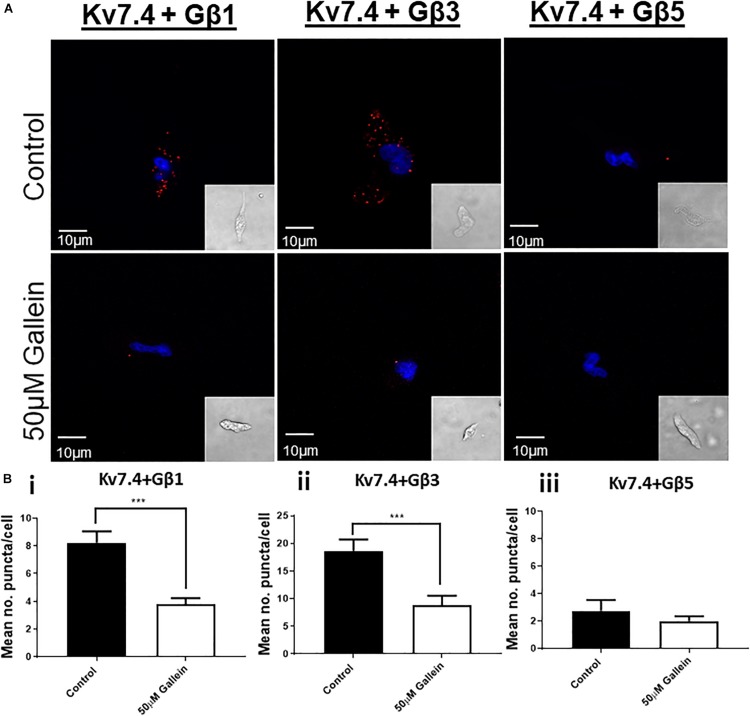
Proximity Ligation Assay detection of Kv7.4 interactions with specific Gβ subunits. **(A)** Representative images of renal artery myocytes investigated for interactions between Kv7.4 and Gβ1, Gβ3 or Gβ5 in control (upper panels) or after treatment with 50 μmol/L gallein (lower panels). **(B)** Mean data of number of PLA puncta/cell before and after 50 μmol/L gallein treatment for Kv7.4- Gβ1 (i, *n* = 30–31, *N* = 5), Kv7.4- Gβ3 (ii, *n* = 15–33, *N* = 3–4), and Kv7.4- Gβ1 (iii, *n* = 11–12, *N* = 2). “*n*” = number of cells and “*N*” = number of animals. Scale bars represent 10 μm. PLA data was analyzed by one-way ANOVA with Bonferroni *post hoc* analysis where *p* < 0.005 is denoted (^∗∗∗^).

To uncover the Gβx subunit(s) responsible for the basal activity of the native vascular Kv7 channel, we first examined the expression of the three Gβ isoforms which enhanced Kv7.4 currents – Gβ1, 3, and 5. Immunofluorescence performed on isolated rat renal artery myocytes showed strong expression of both Gβ1 and 3 in the cytosolic and sub-membranous regions (Figures 4A,B). Interestingly, expression of Gβ5 was confined to the nucleus of these cells (Figure 4C). To ascertain which subunits interacted with Kv7.4 in rat renal myocytes we further undertook a series of PLA studies. It has been shown that proteins can influence ion channels from distances ≤200 nm (Tajada et al., 2017), and PLA detects protein-protein interactions well within this range at ≤40 nm. PLA was performed in rat renal artery myocytes either in control or after treatment with 50 μmol/L gallein. Gallein disrupts Gβγ interactions and so was used as a pharmacological control in determining positive interactions. PLA punctae detected for the combinations of both Kv7.4-Gβ1 (8.2 ± 0.8 puncta/cell) and Kv7.4-Gβ3 (18.7 ± 2 puncta/cell) which were significantly reduced by gallein treatment [change to 3.8 ± 0.4 (Gβ1) and 8.8 ± 1.8 (Gβ3) puncta/cell] (Figure 5). PLA experiments with Kv7.4-Gβ5 PLA antibody combinations did not detect any gallein sensitive PLA puncta (2.7 ± 0.8 puncta/cell to 2.0 ± 0.4 puncta/cell) (Figure 5). These data reveal an association of Kv7.4 with Gβ1 and Gβ3 in vascular smooth muscle cells.

**FIGURE 6:**
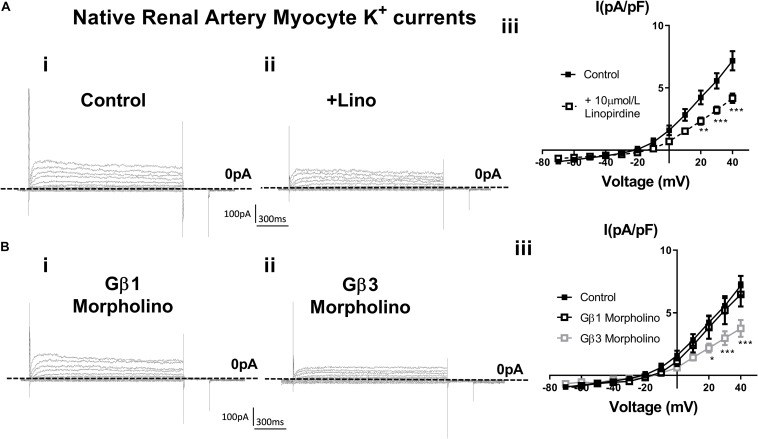
K^+^ currents in renal artery myocytes – effects of linopirdine and β1 or β3 morpholino. **(A)** Representative images of whole cell K^+^ currents recorded in rat renal artery myocytes in control (i) or in the presence of 10 μm/L linopirdine (ii), and mean data [iii, *n* = 17 *N* = 4 (control), *n* = 9 *N* = 4 (linopirdine)]. Zero current is indicated by the dashed line. **(B)** Representative images of whole cell K^+^ currents recorded in rat renal artery myocytes after morpholino knockdown of Gβ1 (i) or Gβ3 (ii) and mean data [iii, *n* = 10 *N* = 4 (Gβ1), *n* = 8 *N* = 4 (Gβ3)]. Zero current is indicated by the dashed line. “*n*” = number of cells and “*N*” = number of animals. Data was analyzed by Bonferroni *post hoc* test following a two-way ANOVA. *p* < 0.05 is denoted (^∗^), *p* < 0.01 is denoted (^∗∗^), and *p* < 0.005 is denoted (^∗∗∗^).

**FIGURE 7:**
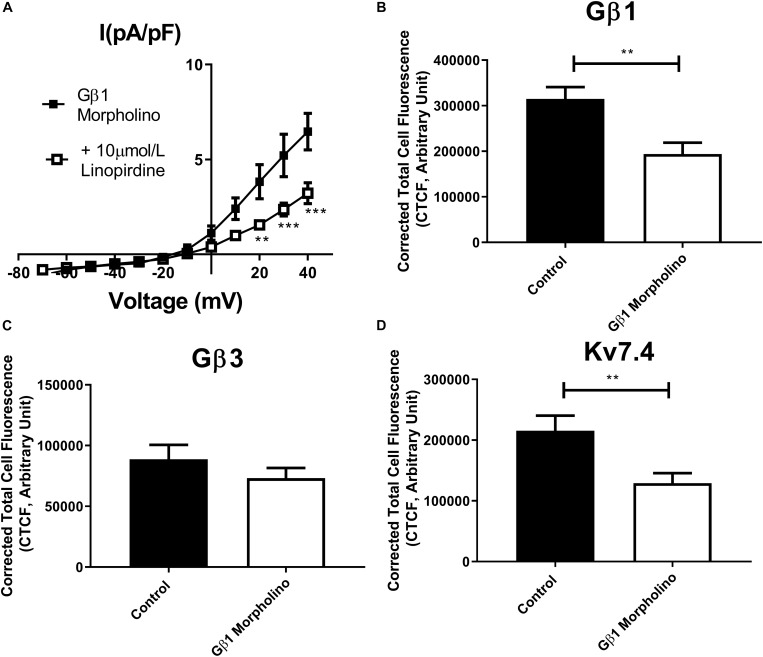
Gβ1 knockdown effects on renal artery myocytes. **(A)** Mean data of whole cell K^+^ currents recorded in rat renal artery myocytes in Gβ1 morpholino in control (*n* = 10, *N* = 4) or in the presence of 10 μm/L linopirdine (*n* = 8, *N* = 4). Mean fluorescence intensity of **(B)** Gβ1 (*n* = 20–22, *N* = 4) **(C)** Gβ3 (*n* = 15, *N* = 3) or **(D)** Kv7.4 (*n* = 14, *N* = 3) in renal artery myocytes in control morpholino or Gβ1 morpholino. “*n*” = number of cells and “*N*” = number of animals. Electrophysiology data was analyzed by Bonferroni *post hoc* test following a two-way ANOVA. IF data was analyzed by one-way ANOVA with Bonferroni *post hoc* analysis. *p* < 0.01 is denoted (^∗∗^) and *p* < 0.005 is denoted (^∗∗∗^).

**FIGURE 8:**
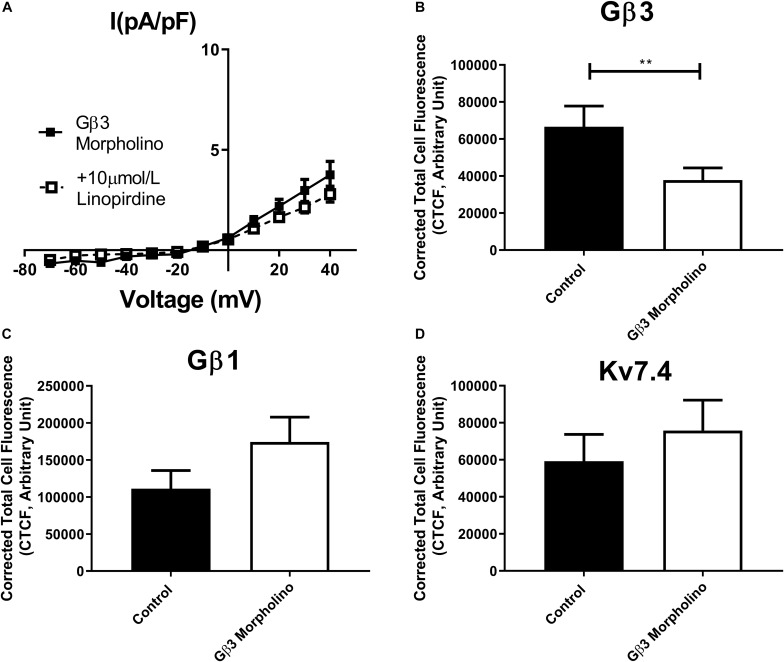
Gβ3 knockdown effects on renal artery myocytes. **(A)** Mean data of whole cell K^+^ currents recorded in rat renal artery myocytes in Gβ3 morpholino in control (*n* = 8, *N* = 4) or in the presence of 10 μm/L linopirdine (*n* = 5, *N* = 4). Mean fluorescence intensity of **(B)** Gβ3 (*n* = 20–23, *N* = 4) **(C)** Gβ1 (*n* = 20–21, *N* = 4) or **(D)** Kv7.4 (*n* = 15, *N* = 3) in renal artery myocytes in control morpholino or Gβ3 morpholino. “*n*” = number of cells and “*N*” = number of animals. Electrophysiology data was analyzed by Bonferroni *post hoc* test following a two-way ANOVA. IF data was analyzed by one-way ANOVA with Bonferroni *post hoc* analysis. *p* < 0.01 is denoted (^∗∗^).

In order to assess the role of Gβ1 and Gβ3 on native vascular Kv7 currents, we performed morpholino directed knockdown of each subunit and examined the Kv7 currents by electrophysiology. Control cells produced K^+^ currents which had a modest sensitivity to the Kv7 channel blocker linopirdine (10 μmol/L) with currents at +40 mV being reduced from 7.2 ± 0.8 pA/pF to 4.2 ± 0.4 pA/pF (Figure 6A). Gβ1 morpholino knockdown cells produced K^+^ currents of the same magnitude as control (6.41 ± 1 pA/pF at 40 mV), whereas K^+^ currents in Gβ3 morpholino knockdown cells were significantly reduced (3.8 ± 0.7 pA/pF at 40 mV) (Figure 6B). Kv7 inhibition with 10 μmol/L linopirdine significantly reduced K^+^ currents in Gβ1 cells (from 6.41 ± 1 pA/pF to 3.2 ± 0.6 pA/pF at 40 mV) (Figure 7A). Immunofluorescence confirmed that Gβ1 morpholino knockdown reduced Gβ1 expression (Figure 7B), and did not affect Gβ3 expression (Figure 7C). However, total Kv7.4 abundance was also reduced in Gβ1 knockdown cells (Figure 7D). In cells treated with Gβ3 morpholino, 10 μmol/L linopirdine had no significant effect on K^+^ currents (from 3.8 ± 0.7 pA/pF to 2.8 ± 0.4 pA/pF at 40 mV) (Figure 8A). Immunofluorescence confirmed knockdown of Gβ3 (Figure 8B), whilst no effects on Gβ1 or Kv7.4 expression was seen (Figures 8C,D).

## Discussion

Several ion channels have now been shown to be regulated by Gβγ subunits revealing the importance of Gβγ subunit-channel regulation in different physiological processes. However, with the increasing recognition of the importance of individual Gβ_*x*_γ_*x*_ combinations in conferring selectivity in effector regulation, defining these properties is a new frontier in Gβγ research. This study demonstrates for the first time the importance of individual Gβ subunits on Kv7.4 channel function. We show that Kv7.4 channel activity can be regulated by several Gβ isoforms in a cell expression system (Gβ1, 3, and 5), but that in native vascular myocytes specific roles for different subunits are described. Gβ1 subunits appear to regulate the synthesis or stability of Kv7.4, whereas Gβ3 regulate basal channel activity in rat renal artery myocytes.

### Gβγ Specificity

With 5 Gβ (1–5) and 12 Gγ (1–5, 7–13) subtypes expressed in mammals, a growing body of evidence has demonstrated specific functions for different subunits, but it remains an oft overlooked aspect of Gβγ regulation. Gβ subunits are highly homologous and between Gβ1, 2, 3, and 4 there are 79–90% sequence similarities (Khan et al., 2013). Gβ5 is the least homologous and shows 51–52% homology when compared to all other subtypes. However, different subunits clearly exert different regulations and here we show specificity in the Gβ isoforms which can regulate Kv7.4 and native vascular Kv7 channels. Only Gβ1, 3, and 5 mirrored the effect on Kv7.4 currents previously reported for purified Gβγ (i.e., increased current amplitude, leftward shift in voltage dependence of activation and increased rate of activation) (Stott et al., 2015). Gβ2 and 4 were without effect. Of the 3 Gβ isoforms that enhanced Kv7.4 channels, only Gβ1 and 3 interacted with Kv7.4 in a gallein-dependent manner in renal artery myocytes. Moreover, only Gβ3 knockdown impaired native Kv7 channel currents analogous to treatment with gallein reported previously (Stott et al., 2015). It is interesting that although Gβ1 enhanced Kv7.4 currents in an overexpression system, knockdown of Gβ1 had no effect on Kv7 currents in renal artery myocytes even though there is a decrease in Kv7.4 protein in these cells. It is likely that the reduction in Kv7.4 is not severe enough to affect overall current density in the acute knockdown protocol over 48 h that we performed. However, our findings clearly indicate that Gβ1 regulates the expression of Kv7.4 at some level (discussed in detail below). Overall the results from our over-expression and native studies suggest that even though Gβ1 can regulate Kv7.4 currents, in myocytes that role is played by Gβ3 and this could indicate that Gβ3 is the preferential regulator of Kv7.4.

Another consideration is that native Kv7 channels present in rat renal artery myocytes could well be a hetero-tetramer with Kv7.5 subunits (Brueggemann et al., 2014; Chadha et al., 2014), and currents produced by these hetero-tetramers are also enhanced with application of Gβγ isolated from bovine brain (unpublished data). It is unclear if differential regulation by different Gβ seen here for Kv7.4 would also apply to the Kv7.4/7.5 channel although it seems likely. It is also known that vascular Kv7 channels undergo regulation by other molecules e.g., KCNE subunits (Jepps et al., 2015). It is possible that in the native system where such complexities exist, these different factors contribute to the Gβ regulation of the channel. Further studies will investigate how different regulators and channel compositions alter Gβ subunit regulation, but it is clear from our study is that Gβ3 basally regulates the native Kv7 current in renal artery myocytes. This is of interest in the area of the vascular biology of hypertension, where a polymorphism in Gβ3 has been associated with a hypertensive phenotype (Semplicini et al., 2015). This polymorphism produces a truncated version of the Gβ3 protein, but it remains to be seen how this might regulate Kv7.4 or native vascular Kv7 channels. Finally, the role of Gβ5 remains unclear as we did not find this to be associated with Kv7.4 in rat renal artery myocytes, but it is possible that this may be an important regulator of Kv7.4 channels in other cell types.

It is important to note that our study has not investigated the role of Gγ in Kv7.4 channel regulation and this could well be an important determinant as described recently for Kir channels (Tabak et al., 2019). Certainly, in overexpression studies the Gγ natively expressed in these cells could be a determinant in the behavior of the Gβ subunits expressed. Subunits have been shown to exhibit preferences in forming Gβ_*x*_γ_*x*_ dimers (Pronin and Gautam, 1992; Schmidt et al., 1992; Dupre et al., 2009) whereas Gβ5 is thought to preferentially couple not to Gγ, but to a Regulator of G protein Signaling (RGS7) (Cabrera et al., 1998). There have also been differing reports about the coupling of Gβ3, with some reports showing that it can function without Gγ (Schmidt et al., 1992; Dingus et al., 2005; Poon et al., 2009). This is likely due to differences in experimental methods used, but what is clear is that what can theoretically interact *in vitro* may not be what actually occurs *in situ*. Gγ subunits are much more diverse, and some have argued that the diversity in Gβγ signaling is due to the Gγ in the dimer, rather than the Gβ. However our studies are complemented by work in native cells and we clearly show definitive roles for Gβ1 and 3 here and we demonstrate that disabling this subunit affects Kv7 channels. However, further studies will begin to examine the role of Gγ in these processes.

### Gβγ in Trafficking

We describe here that knockdown of Gβ1 decreases Kv7.4 abundance in renal artery myocytes. This was an unexpected finding from a control experiment, but displays another different facet of Gβ regulation. As discussed above, the decrease in Kv7.4 was not severe enough to affect Kv7 current density, but it is possible that a longer period of knockdown would give more information on this relationship. Knockout of Gβ1 has been shown to be embryonic or perinatally lethal so this is not possible (Okae and Iwakura, 2010). However, these findings are supported by previous findings where Gβ1 regulate protein expression – knockdown increases Gβ4 expression (Hwang et al., 2005) and also alters Kir expression levels (Zylbergold et al., 2014). As it stands it is impossible to know at what level Gβ1 regulates Kv7.4 expression in myocytes without a much more detailed investigation into Kv7.4 protein synthesis and stability in these cells, but insights from previous work shows us that Gβγ can variously be involved in transcription, translation, trafficking and degradation of proteins and their genes (Khan et al., 2016). Knockdown studies have also demonstrated that Gβ subunits are involved in the expression of Gγ subunits – Gβ1 knockdown results in decreased Gγ5 expression, whilst Gγ2, 5, and 12 were decreased by Gβ2 knockdown (Hwang et al., 2005) – but this may be not be a direct regulatory effect but due to a result of removing the binding partners of these Gγ subunits ceases their production.

These findings underlie how the identity of the Gβ subunit is crucial for different aspects of ion channel regulation. That we demonstrate that two closely related Gβ subunits have two different regulatory capacities for Kv7.4 channels in the vasculature is quite striking, and is important in changing the way we think about Gβγ regulation. This work is significant in the growing move away from viewing Gβγ as homogenous dimer pairs to viewing these subunits as complex, disparate entities with specific regulatory responsibilities within cells.

## Materials and Methods

### Ethical Approval

All experiments were performed in accordance with the United Kingdom Animals (Scientific Procedures) Act 1986.

#### Cell Culture

Chinese Hamster Ovary (CHO) cells were maintained in Dulbecco’s modified Eagle’s medium – high glucose solution supplemented with 10% fetal bovine serum and 1% penicillin/streptomycin in an incubator with 5% CO_2_. Cells were split into six well plates for transfection for electrophysiology experiments. Cells were transfected with Kv7.4 and either Gβ1–5 or an empty vector (EV). Plasmids were mixed with Lipofectamine 2000 in Opti-MEM for 20 min before being added to cells. A total of 3 μg of plasmid was transfected in a 1:1 ratio for 24 h. Cells were briefly trypsinised on the day of experiments and plated on 13 mm coverslips in media at room temperature for 30 min and were then stored in the fridge for use within 8 h.

#### Animals

Male Wistar rats (175–225 g, Charles River United Kingdom) were kept in a 12 h light/dark cycle with free access to food and water. Animals were culled on the day of experiment by Schedule 1 cervical dislocation. Renal arteries (RA) were dissected of adherent fat and connective tissue and stored on ice in a physiological saline solution (PSS) containing (in mmol/L); 4.5 KCl, 120 NaCl, 1.2 MgSO_4_.7H_2_O, 1.2 NaH_2_PO4.2H_2_O, 25 NaHCO_3_.5 D-Glucose, and 1.25 CaCl_2_.

#### Morpholino Knockdown

To study the role of Gβ1 and Gβ3 in native Kv7 currents, knockdown in RA was performed by transfection with morpholino nucleotides as described in vessels previously (Stott et al., 2018). Gβ1 and Gβ3 morpholino nucleotides and mismatched control nucleotides (5 μmol/L, Genetools) were mixed with Lipofectamine 2000 (Life Technologies, United Kingdom) in Opti-MEM and left at room temperature for 2 h. RA were transfected with this mix in Dulbecco’s modified eagle’s medium F-12 with 1% penicillin/streptomycin at 37°C for 48 h.

#### Cell Isolation

Freshly dissected renal arteries were used for isolation of individual myocytes for proximity ligation assay, whereas vessels incubated in control or morpholino for 48 h were used for electrophysiology. Vessels were bathed for 10 min in a nominally Ca^2+^ free solution (in mmol/L: 6 KCl, 120 NaCl, 1.2 MgCl2, 12 D-glucose and 10 HEPES, pH 7.4 with NaOH). Vessels were then incubated at 37°C for 20 (incubated) or 23 (fresh) min in Ca^2+^ free solution containing in mg/ml: 1.5 collagenase, 0.75 thermolysin, 1 trypsin inhibitor and 1 bovine serum albumin (all Sigma Aldrich, United Kingdom). Vessels were then washed in Ca^2+^ free solution for 10 min and then triturated to liberate myocytes. The cell solution was plated on 13 mm coverslips in a 24 well plate and supplemented with an equivalent volume of 2.5 mmol/L Ca^2+^ solution to allow the cells to adhere.

#### Immunofluorescence

Freshly isolated myocytes from renal arteries from vessels transfected with control or Gβ1 and Gβ3 morpholino were prepared as above. Cells were fixed with 3% PFA on ice for 20 min and stored in PBS at 4°C. Cells were treated with 0.1 mol/L glycine for 5 min and then incubated in blocking solution (PBS containing 0.1% Triton X-100 and 1% bovine serum albumin) for 1 h at RT before being incubated overnight at 4°C with the following primary antibodies: (i) rabbit anti-Kv7.4 antibody (dilution 1:100, Abcam, Cambridge, United Kingdom); (ii) rabbit anti-Gβ1 (dilution 1:100, Genetex, #GTX114442); mouse anti-Gβ3 (dilution 1:100, Abnova #H00002784-MO1) (iii) rabbit anti- Gβ5 (dilution 1:200, Abcam, #ab185206). Samples were then washed with PBS and incubated for 1 h with donkey anti-rabbit or donkey anti-mouse secondary antibodies conjugated to Alexa Fluor 568, respectively (dilution 1:100, Thermo-Fisher, Paisley, United Kingdom). All antibodies were diluted in blocking solution. Subsequently, samples were washed with PBS and mounted using VECTASHIELD Antifade Medium containing DAPI for nuclei counterstaining (Vector Laboratories, Peterborough, United Kingdom). Coverslips were analyzed using a Zeiss LSM 510 Meta argon/krypton laser scanning confocal microscope (Carl Zeiss, Jena, Germany). Corrected total cell fluorescence (CTCF) was calculated using ImageJ software as elsewhere described (Burgess et al., 2010).

#### Proximity Ligation Assay

Freshly isolated cells were used as described above. 1 mL of solution containing 2.5 mmol/L CaCl_2_ was added to each well and cells were placed in an incubator (37°C, 5% CO_2_) for 30 min to equilibrate. Cells then underwent treatment: 50 μmol/L gallein or DMSO control (30 min). Gallein was used as a pharmacological control for identifying positive interactions with Gβx subunits, and only interactions which were sensitive to gallein were considered as true interactions. Coverslips were immediately fixed with 3% PFA on ice for 20 min and stored in PBS at 4°C. For the proximity ligation assay, cells were permeablised with 0.01% Triton X-100 for 5 min. The Duolink *in situ* PLA detection kit (Sigma-Aldrich, United Kingdom) was used to detect single molecule interactions for Kv7.4 (anti-mouse (N43/6, RRID:AB_2131828, UC Davis/NIH NeuroMab Facility) or anti-rabbit (Abcam, Cambridge, United Kingdom) and G protein β subunits; anti-Gβ1 (rabbit, Genetex, #GTX114442), anti-Gβ2 (rabbit, Cusabio, #CSB-PA009604ESR2HU), anti-Gβ3 (mouse, Abnova #H00002784-MO1), and anti-Gβ5 (rabbit, Abcam, #ab185206). Experiments were performed as per manufacturer’s instructions; primary antibodies were incubated at 1:200 overnight at 4°C. Red fluorescent oligonucleotides produced as the end product of the procedure were visualized using a Zeiss Confocal LSM 510. Images were analyzed using ImageJ software using the particle detector tool. The number of puncta per cell was calculated as the average of two mid sections in each cell.

#### Electrophysiology

All current recordings were made using Axopatch 200B amplifier (Axon Instruments) at room temperature. Whole –cell electrical signals were generated and digitized at 1 kHz using a Digidata 1322A hosted by a PC running pClamp 9.0 software (Molecular Devices). For recordings cells were placed in an external solution containing (in mmol/L); 140 NaCl, 4 KCl, 2 CaCl_2_, 1 MgCl_2_, and 10 HEPES. Patch pipettes with a resistance of 4–12 MΩ were filled with a pipette solution containing (in mmol/L); 110 K gluconate, 30 KCl, 0.5 MgCl_2_, 5 HEPES, and 0.5 EGTA. Cells were held at −60 mV and currents amplitude was monitored by application of a test pulse to 40 mV every 20 s. To generate current-voltage relationships a voltage step protocol was used from a holding potential of −60 mV testing a range of voltages from −70 to 40 mV in 10 mv increments at 15 s intervals.

#### Reagents

Gβ1 plasmid was kindly gifted by Dr. Kim Jonas. Gβ2–5 were purchased from Addgene (catalog nos. 36113, 36114, 54469, and 54470). Gallein and linopirdine were obtained from Tocris (Bristol, United Kingdom). Gβγ subunits isolated from bovine brain were purchased from Merck Millipore.

### Statistics

All values are expressed as mean ± SEM. All statistical analyses and non-linear regression curve fitting were performed with GraphPad Prism 7 software. In experiments which utilize native rat myocytes “n” represents the number of cells analyzed and “N” represents the number of subjects. Analyzes of immunofluorescence data were performed by Student’s *t*-test, and Proximity Ligation Assay data by one-way ANOVA multiple comparisons test with Bonferroni *post hoc* analysis. Analyses of electrophysiological data used two-way ANOVA with Bonferroni *post hoc* analysis. Differences were considered significant at *p* < 0.05.

## Data Availability Statement

The datasets generated for this study are available on request to the corresponding author.

## Ethics Statement

The animal study was reviewed and approved by the St George’s University of London Animal Ethics Committee.

## Author Contributions

JS undertook the research, analyzed and interpreted the data, and wrote the manuscript. JS and IG designed and funded the data and revised the manuscript. Both authors read and finally approved the manuscript.

## Conflict of Interest

The authors declare that the research was conducted in the absence of any commercial or financial relationships that could be construed as a potential conflict of interest. The handling Editor declared past co-authorship with one of the authors IG.
